# A 3-MicroRNA Signature Identified From Serum Predicts Clinical Outcome of the Locally Advanced Gastric Cancer

**DOI:** 10.3389/fonc.2020.00565

**Published:** 2020-06-19

**Authors:** Shangxiang Chen, Jiawen Lao, Qirong Geng, Ji Zhang, Aiwen Wu, Dazhi Xu

**Affiliations:** ^1^Department of Gastric Surgery, Sun Yat-sen University Cancer Center, Guangzhou, China; ^2^State Key Laboratory of Oncology in South China and Collaborative Innovation Center for Cancer Medicine, Guangzhou, China; ^3^Department of Hematology Oncology, Sun Yat-sen University Cancer Center, Guangzhou, China; ^4^Department of Neurosurgery, Sun Yat-sen University Cancer Center, Guangzhou, China; ^5^Key Laboratory of Carcinogenesis and Translational Research (Ministry of Education), Department of Gastrointestinal Surgery, Peking University Cancer Hospital and Institute, Beijing, China

**Keywords:** microRNA, survival, advanced gastric cancer, serum, prognosis

## Abstract

**Background:** Current staging systems are inadequate for evaluating the prognosis of patients with locally advanced gastric cancer (LAGC, stages II–III). Therefore, we developed a serum microRNA (miRNA) signature to facilitate individualized management of these patients.

**Methods:** Using microarray analysis, we analyzed 12 serum specimens based on different prognoses (good survival group, *n* = 7; poor survival group, *n* = 5). We identified and confirmed differential expression of these miRNAs using quantitative reverse transcription PCR (qRT-PCR) of serum from 51 patients with LAGC. A three miRNA-based classifier was established as a training set by Cox proportional hazard regression and risk-score analysis. We validated the prognostic accuracy of this model in an internal validation cohort (Sun Yat-Sen University Cancer Center, SYSUCC validation cohort, *n* = 50) and an external independent cohort (Beijing Cancer Hospital, BJCH cohort, *n* = 67).

**Results:** Three miRNAs were found to be associated with survival of LAGC (*P* < 0.001 for miR-132, *P* = 0.011 for miR-548a-3p, and *P* < 0.001 for miR-1826). A three-miRNA signature was developed for the training set, and a significant difference was found between the survival of low- and high-risk score patients (*P* < 0.01). The combination of the miRNA signature and tumor–node–metastasis (TNM) stage exhibited superior discrimination. Consistent results were obtained by further validation of the internal set and the BJCH set, which confirmed the predictive value of the model.

**Conclusions:** We built an easy-to-use prognostic signature using three serum miRNAs as markers. Our miRNA signature may improve postoperative risk stratification and serve as a complement to the TNM staging system.

## Introduction

Despite the declining incidence of gastric cancer (GC), it remains the second leading cause of cancer-related death in the world (1, 2). For locally advanced GC (LAGC, stages II–III), gastrectomy with D2 lymphadenectomy combined with adjuvant chemotherapy is the standard treatment, especially in Asia (3). Recently, several studies of neoadjuvant chemotherapy/chemoradiotherapy have reported promising results for LAGC patients, who have a high risk of metastasis (3). However, many patients still exhibit very poor prognosis (3). Given that conventional assessment approaches do not clearly distinguish between patients with a high or low risk of metastasis, it is a major challenge to identify more effective survival prediction methods.

Advances in microRNA (miRNA) expression profiling provide probabilities for tumor prognosis prediction and treatment design. Extensive studies have reported that miRNAs are involved in tumor development, differentiation, and pathogenesis (4–7). It has been suggested that miRNA profiles may be a good alternative to expression profiles of protein-coding genes in tumor classification and prediction (8). Moreover, a model integrating multiple biomarkers could improve predictive efficiency (9, 10). Therefore, miRNA expression profiling could provide more accurate biological information compared with protein-coding gene profiling (11, 12).

In this study, we aimed to develop a reliable, non-invasive serum miRNA signature with prognostic value for LAGC patients.

## Materials and Methods

### Serum Patients and Tumor RNA Samples

Between January 2002 and October 2008, 113 patients with LAGC at the Cancer Center of Sun Yat-sen University (SYSUCC) (Guangzhou, China) were included in this study. Blood samples from patients who underwent D2-lymphadenectomy were obtained for prognostic miRNA signature establishment and internal validation. Patients with stage II and III GC having complete clinicopathological records were included. Patients were staged according to the 7th American Joint Committee on Cancer (AJCC) tumor–node–metastasis (TNM) staging system (13). None of the patients had received previous treatment with any anticancer therapy prior to surgery. Twelve cases were selected for miRNA microarray analysis. Seven patients who had survival times >60 months (average, 76 months; range, 65–94 months) were identified as the good survival group, whereas five patients who survived <25 months (average, 7 months; range, 3–12 months) were identified as the poor survival group. The remaining 101 cases were randomly classified as either training (*n* = 51) or testing (*n* = 50) sets using computer-generated random numbers. For further external validation, another independent cohort (*n* = 67) from Beijing Cancer Hospital (BJCH) (Beijing, China) was enrolled between November 1, 2008 and May 1, 2010. The process of the study is shown in the flow chart (Figure S5). Pathological diagnosis of GCs was confirmed by at least two professional pathologists. Ethical approval was obtained from both Sun Yat-sen University Cancer Center and Beijing Cancer Hospital research ethics committees.

### MiRNA Expression Profiling

Microarray was performed on 12 serum samples miRNA expression profile by CapitalBio (CapitalBioCorp). Procedures are described in detail on the CapitalBio website (http://www.capitalbio.com). Briefly, procedures included total RNA extraction, sample quality control, miRNA isolation using MirVana miRNA isolation Kit (Ambion), FlashTagTM Biotin Labeling of miRNAs (Genisphere), hybridization to Affymetrix GeneChip miRNA microarray (Affymetrix), microarray washing, staining, and scanning. When miRNA expression changed by at least ±2-fold and *P* < 0.05, miRNAs were considered significantly differentially expressed and selected for quantitative PCR (qPCR) validation. Clustering analysis was performed by Cluster 3.0-based primarily on fold change >1.50 (14).

### qRT-PCR Analysis of miRNAs

Quantitative reverse transcription PCR (qRT-PCR) was performed on selected miRNA candidates to validate miRNA array results. Total RNA was extracted from 200 μl of serum using TRIzol LS reagent. Briefly, 600 μl of TRIzol® LS was added into 200 μl of serum. Samples were mixed with a pipette and incubated for 5 min at room temperature. Chloroform (160 μl) was added to samples and shaken vigorously by hand for 15 s, followed by incubation for 10 min at room temperature. Mixtures were centrifuged at 12,000 *g* for 15 min at 4°C, and 500 μl of the upper aqueous phase was placed into a new tube. An equal volume of 100% isopropanol was added to the aqueous phase and incubated at room temperature for 10 min, followed by centrifugation at 12,000 *g* for 10 min at 4°C to pellet the RNA. The supernatant was discarded, and the RNA pellets were washed with 1 ml 75% ethanol diluted in RNase-free H_2_O. The RNA pellet was vortexed and centrifuged at 7,500 *g* for 5 min at 4°C, dried for 10 min at room temperature, and dissolved in 20 μl of RNase-free H_2_O.

The expression levels of miRNAs were normalized and quantified to the small nuclear U6 RNA, which was used as an internal control as previously reported (15). After the reaction, cycle threshold (CT) values were determined based on fixed threshold settings. To calculate the expression levels of miRNA, the standard curve of each miRNA was prepared. All reactions, including no-template controls, were run in triplicate. Data were analyzed using the 2^−ΔΔ*Ct*^ method. Primer sequences of the miRNAs are shown in Table S2.

### Statistical Analysis

The primary set of patients (*n* = 101) was randomly assigned as either training set (*n* = 51) or testing set (*n* = 50). In the training set, we used univariate and multivariate Cox regression analysis to evaluate the association between expression levels of miRNAs and survival (16). MiRNAs with hazard ratio (HR) for death <1 were defined as protective miRNAs, while those HRs >1 were considered risk miRNAs. For miRNAs that significantly correlated with survival, we assigned each patient a risk score calculated by a liner combination of the expression level of the miRNA weighted by the regression coefficient (17). To further test the predictive value of the risk score, the same algorithm was validated in the testing and the BJCH sets.

Comparisons between two groups were completed using *t* test for continuous variables and chi-square test for categorial variables. The Kaplan–Meier method was used for the analysis between variables and survival, and log-rank test was used for comparing survival curves. Cox regression models were used for univariable and multivariable survival analysis and Cox regression coefficients for generating the risk score classifier. Akaike's information criterion (AIC) method and receiver operating characteristic (ROC) curves were used for evaluating prognostic or predictive accuracy. Higher area under the curve (AUC) values and lower AIC values indicate better discrimination. All statistical analyses were performed using the software statistical package for social sciences version 20.0 (SPSS, Chicago, IL). Results were considered statistically significant when *P* < 0.05.

### MiRNA-Targeted Gene Prediction and Signal Pathway Analysis

To identify possible target genes of these three differently expressed miRNAs, we integrated the predicted genes from TargetScan (http://www.targetscan.org), microRNAdb (http://bioinfo.au.tsinghua.edu.cn/micrornadb/index.php), miTARbase (http://mirtarbase.mbc.nctu.edu.tw/php/index.php), and miRPathDB (https://omictools.com/mirna-pathway-dictionary-database-tool). The online function annotation tool DAVID (https://david.ncifcrf.gov/summary.jsp) was used to annotate the molecular function of target genes and signaling pathways with which they are involved.

## Results

### Expression Profiles of MicroRNAs

**Figure 1:**
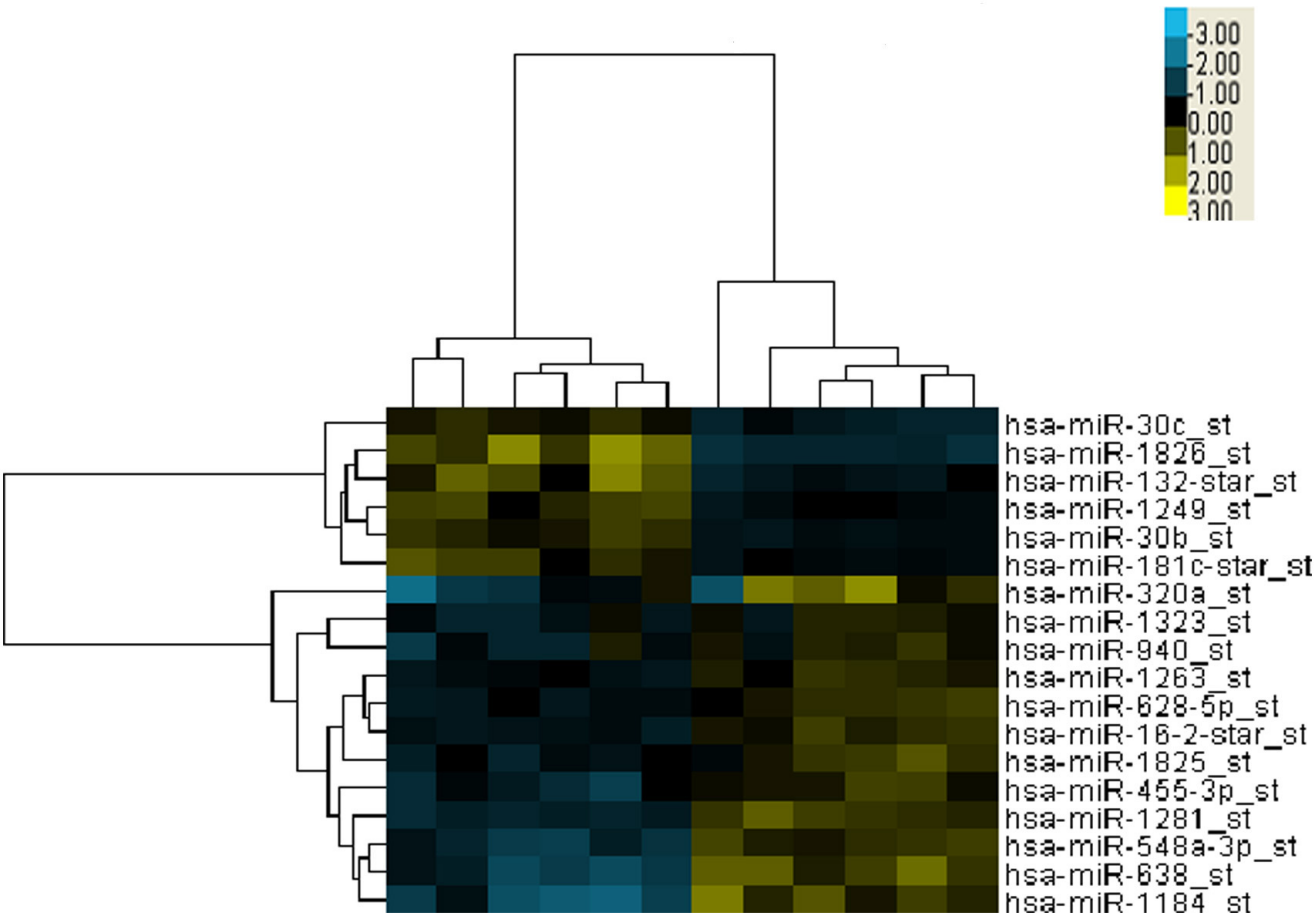
Cluster analysis of expressed microRNAs (miRNAs) in 12 selected locally advanced gastric cancer (LAGC) serum samples. Five miRNAs were identified as dysregulated, including two upregulated in the good survival group and three downregulated in the poor survival group. The criteria of fold change >1.5 was used. Columns represent the samples, and rows represent the miRNAs (blue, black, and yellow represents downregulated, unchanged, and upregulated, respectively).

To identify differentially expressed microRNAs between the good and poor survival groups, microarrays of 12 patients' serum specimens were performed to detect relative expression levels of miRNAs (Table S1). We identified two microRNAs that were upregulated more than 2-fold (miR-1826 and miR-132) in the good survival group compared with three miRNAs (miR-548a-3p, miR-638, and miR-1184) that were downregulated more than 2-fold in the good survival group (Table 1). Hierarchical clustering analysis using microRNA fold change >1.50 showed distinct expression patterns between the two groups (Figure 1).

**Table 1:** Relative expression levels of serum miRNAs from the 12 selected patients.

**miRNAs deregulated (*P* < 0.001)**	**Long survival vs. short survival**	
	**Direction**	**Fold change**
miR-1826	Up	3.15
miR-132	Up	2.03
miR-548a-3p	Down	0.48
miR-638	Down	0.44
miR-1184	Down	0.34

*miR, microRNA*.

*microRNAs dysregulated more than 2-fold in good survival group were identified*.

### Three Serum MicroRNA Classifiers and Survival

To further examine if these microRNAs can be used for outcome prediction, the primary set was randomly assigned to training and testing samples to identify prognostic factors. There were no significant differences in clinicopathological features between the two sets (all *P* > 0.05). The characteristics of the training, testing, and BJCH sets are summarized in Table 2. To explore the relationship between miRNAs and overall survival, we used the Youden index method to generate optimal cutoff points for the five candidate miRNAs in the training data set (18). Three miRNAs (miR-1826, miR-132, and miR-548a-3p) from these five miRNAs were identified by Cox proportional hazard regression. Expression levels of the three miRNAs were significantly associated with patient survival (miR-1826: 95%CI, 0.121–0.529, *P* < 0.001; miR-132: 95%CI, 0.118–0.517, *P* < 0.001; and miR-548a-3p: 95%CI, 1.233–5.069, *P* = 0.011). High expression levels of miR-548a-3p (median, 314) or low expression levels of miR-1826 (median, 1,416) and miR-132 (median, 5.9) predicted unfavorable survival. Differences in median survival with respect to different expression levels of these three miRNAs were statistically significant (*P* < 0.001 for miR-132, *P* = 0.008 for miR-548a-3p, and *P* < 0.001 for miR-1826) (Table 3). Interestingly, similar conclusions were reached in the testing, primary, and BJCH sets when the same threshold values were applied (data not shown). Multivariate Cox regression was then used to build a prognostic model using the three miRNAs selected in the training set. A formula was then derived to calculate the risk score for each patient's overall survival according to the expression levels of these three miRNAs. Based on individual regression coefficients, a risk score model for outcome prediction was built as follows: risk score = (2.057^*^miR-548a-3p) + (−0.364^*^expression level of miR-132 expression level) + (−0.350^*^expression level of miR-1826). In this formula, the plus sign equals a risk factor, and the minus sign equals a protective factor. A higher risk score indicates worse overall survival.

**Table 2:** Clinical features of the training set, testing set, and BJCH set.

**Characteristics**	**SYSUCC**		*P* value*	**BJCH set (*n* = 67)**
	**Training set (n = 51)**	**Testing set (n = 50)**		
Gender			0.757	
Male	36	36		47
Female	15	14		20
Age (Years)			0.906	
Median	56	59		57
Range	27–77	20–82		26–73
Tumor location			0.805	
Upper	20	23		15
Middle	7	6		13
Lower	24	21		39
Differentiated Type			0.135	Unknown
Well and moderately differentiated (Differentiated)	10	7		
Poorly differentiated (Undifferentiated)	41	43		
AJCC stage			0.272	
II	9	14		21
III	42	36		46
Median survival time (months)	22	33	0.418	42.59

* *p value performed by log-rank test represents the statistical significance between the training set and testing set*.

**Table 3:** miRNA level and survival of GC patients in training set.

	**Patients**	**Deaths**	**MST (months)**	***P* value^a^**	**95%CI**
**miR-132**					
No. of patients	51	33			
Low, ≤ 5.9	26	22	7.78		
High, >5.9	25	11	62.00	<0.001	0.247 (0.118, 0.517)
**miR-548a-3p**					
No. of patients	51	33			
Low, ≤ 314	26	13	46		
High, >314	25	20	13	*P* = 0.011	2.499 (1.233, 5.069)
**miR-1826**					
No. of patients	51	33			
Low, ≤ 1416	26	22	7.78		
High, >1416	25	11	62.00	<0.001	0.253 (0.121, 0.529)

*GC, Gastric Cancer; MST, median survival time*.

a *Cox proportional hazards model*.

### Three Serum MicroRNA Classifiers Predict LAGC Survival

**Figure 2:**
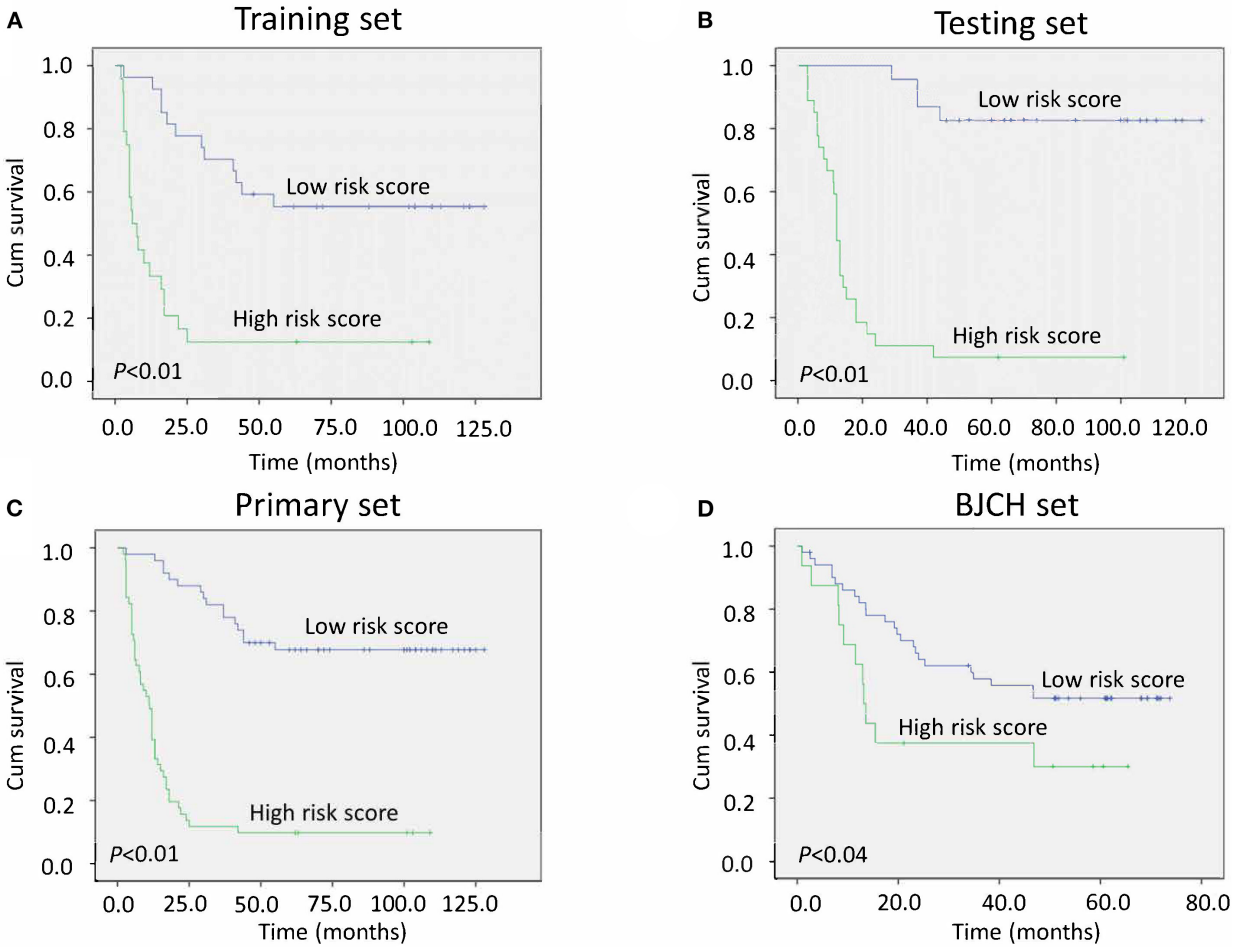
Kaplan–Meier curves of gastric cancer (GC) patients indicate the relationship between risk score and clinical outcome. **(A)** Survival curves of low-risk scores compared with high-risk scores in the training set. **(B)** Survival curves of low-risk scores compared with high-risk scores in the testing set. **(C)** Survival curves of low-risk scores compared with high-risk scores in the primary set. **(D)** Survival curves of low-risk scores compared with high-risk scores in the Beijing Cancer Hospital (BJCH) set.

To validate whether the three miRNA classifiers could predict survival of LAGC patients, we classified patients into good or poor survival groups using the median risk score as the cutoff point. Kaplan–Meier analysis and log-rank test indicated that the two groups had significantly different overall survival. The low-risk score group exhibited better survival compared with the high-risk score group (median survival time, 80.00 vs. 7.78 months, respectively) (*P* < 0.01) (Figure 2A). To further validate whether this classifier was applicable to other cohorts, we analyzed the survival of the testing, primary, and BJCH sets, and the results were consistent with those of the training set. Indeed, high-risk score values increased the possibility of shortened survival more than the low-risk score values (median survival: low- vs. high-risk score groups are 70.00 vs. 12.00 months for the testing set, 70.00 vs. 11.15 months for the primary set, and 50.85 vs. 13.56 months for the BJCH set) (*P* < 0.05) (Figures 2B–D). Furthermore, elevated HRs indicated worse survival referred to a higher risk score (5.00 for the training set, 10.45 for the testing set, 7.73 for the primary set, and 2.28 for the BJCH set) (Table 4).

**Table 4:** Risk score and survival of GC patients in training set, testing set, primary cohort, and BJCH set.

**Data set**	**Patients**	**Deaths**	**MST (months)**	***P* Value^a^**	**95%CI**
**Training set**					
No. of patients	51	33			
Low risk, ≤ 92.327	25	10	70.00		
High risk, >92.327	26	23	7.78	<0.001	5.000 (2.327, 10.743)
**Testing set**					
No. of patients	50	29			
Low risk, ≤ 92.327	25	6	70.00		
High risk, >92.327	25	23	12.00	<0.001	10.450 (4.073, 26.810)
**Primary set**					
No. of patients	101	62			
Low risk, ≤ 92.327	50	16	70.00		
High risk, >92.327	51	46	11.15	<0.001	7.726 (4.262, 14.004)
**BJCH set**					
No. of patients	67	35			
Low risk, ≤ 92.327	51	24	50.85		
High risk, >92.327	16	11	13.56	*P* = 0.038	2.28 (1.046, 4.943)

*GC, Gastric Cancer; MST, median survival time*.

a *Cox proportional hazards model*.

**Figure 3:**
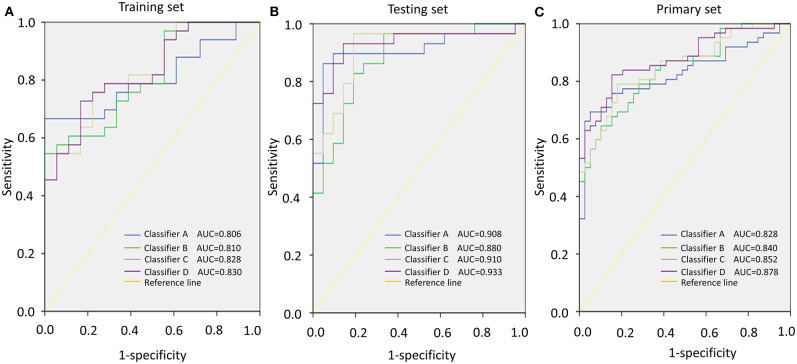
Area under the curve (AUC) according to different classifiers (classifiers A–D). **(A)** AUCs of classifiers A–D in the training set. **(B)** AUCs of classifiers A–D in the testing set. **(C)** AUCs of classifiers A–D in the primary set.

To verify whether this classifier was more specific to discriminate LAGC than other combinations, we combined miRNAs into classifier A (miR-132 and miR-1826), classifier B (miR-132 and miR-548a-3p), classifier C (miR-548a-3p and miR-1826), and classifier D (miR-132, miR-548a-3p and miR-1826) groups. Then, these four classifiers were applied to training, testing, and primary sets. In the training set, classifier D significantly distinguished the good and poor survival groups (*P* < 0.001) and increased the AUC to 0.830, which was higher than the other three classifiers. The specificity was 0.833, and sensitivity was 0.727. Similar results were found in the testing/primary sets. In total, our classifier increased the specificity and sensitivity more so than either other combined classifiers or individual miRNAs, exhibiting the highest AUC value (Table 5, Figure 3).

**Table 5:** Comparison of the AUCs for the different classifiers.

	**AUC**	**95% CI**	***P* Value**
**Training Set**			
Classifier A	0.806	(0.689–0.924)	<0.001
Classifier B	0.810	(0.693–0.927)	<0.001
Classifier C	0.828	(0.717–0.940)	<0.001
Classifier D	0.830	(0.719–0.941)	<0.001
**Testing set**			
Classifier A	0.908	(0.819–0.997)	<0.001
Classifier B	0.880	(0.785–0.975)	<0.001
Classifier C	0.910	(0.824–0.996)	<0.001
Classifier D	0.933	(0.858–1.000)	<0.001
**Primary set**			
Classifier A	0.828	(0.747–0.908)	<0.001
Classifier B	0.840	(0.765–0.914)	<0.001
Classifier C	0.852	(0.779–0.924)	<0.001
Classifier D	0.878	(0.812–0.943)	<0.001

*classifier A, miR-132 and miR-1826; classifier B, miR-132 and miR-548a-3p; classifier C, miR-548a-3p and miR-1826; classifier D, miR-132, miR-548a-3p and miR-1826*.

*AUC, area under curve*.

### Three Serum MicroRNA Classifiers and TNM Staging

**Figure 4:**
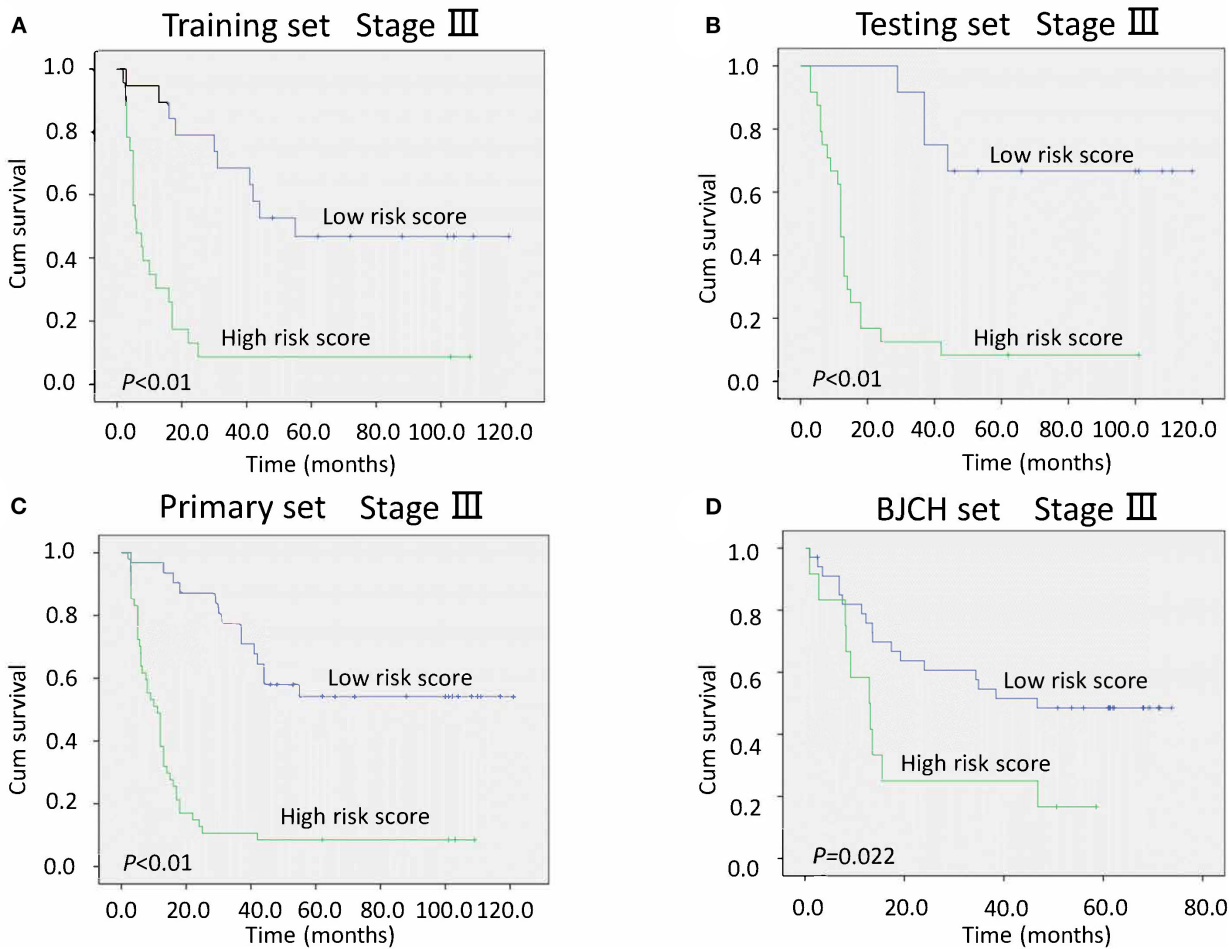
Kaplan–Meier curves of stage III gastric cancer (GC) patients indicate the relationship between risk score and clinical outcome. **(A)** Survival curves of low-risk scores compared with high-risk scores in the training set. **(B)** Survival curves of low-risk scores compared with high-risk scores in the testing set. **(C)** Survival curves of low-risk scores compared with high-risk scores in the primary set. **(D)** Survival curves of low-risk scores compared with high-risk scores in the Beijing Cancer Hospital (BJCH) set.

After adjustment for clinicopathological variables, the risk score and TNM stage remained powerful and independent prognostic indicators of overall survival in the primary set (*P* < 0.01 for risk score and *P* = 0.021 for TNM stage). We further stratified patients by risk score in stages II and III. Notably, low-risk score groups exhibited better survival than the high-risk score groups in stage III (all *P* < 0.05, Figure 4). However, different from stage III, no statistical significance was found between low- and high-risk score groups in either primary or BJCH sets in stage II (*P* > 0.05, Figure S1). The reason for this may be the small sample size of patients with stage II in this study.

**Figure 5:**
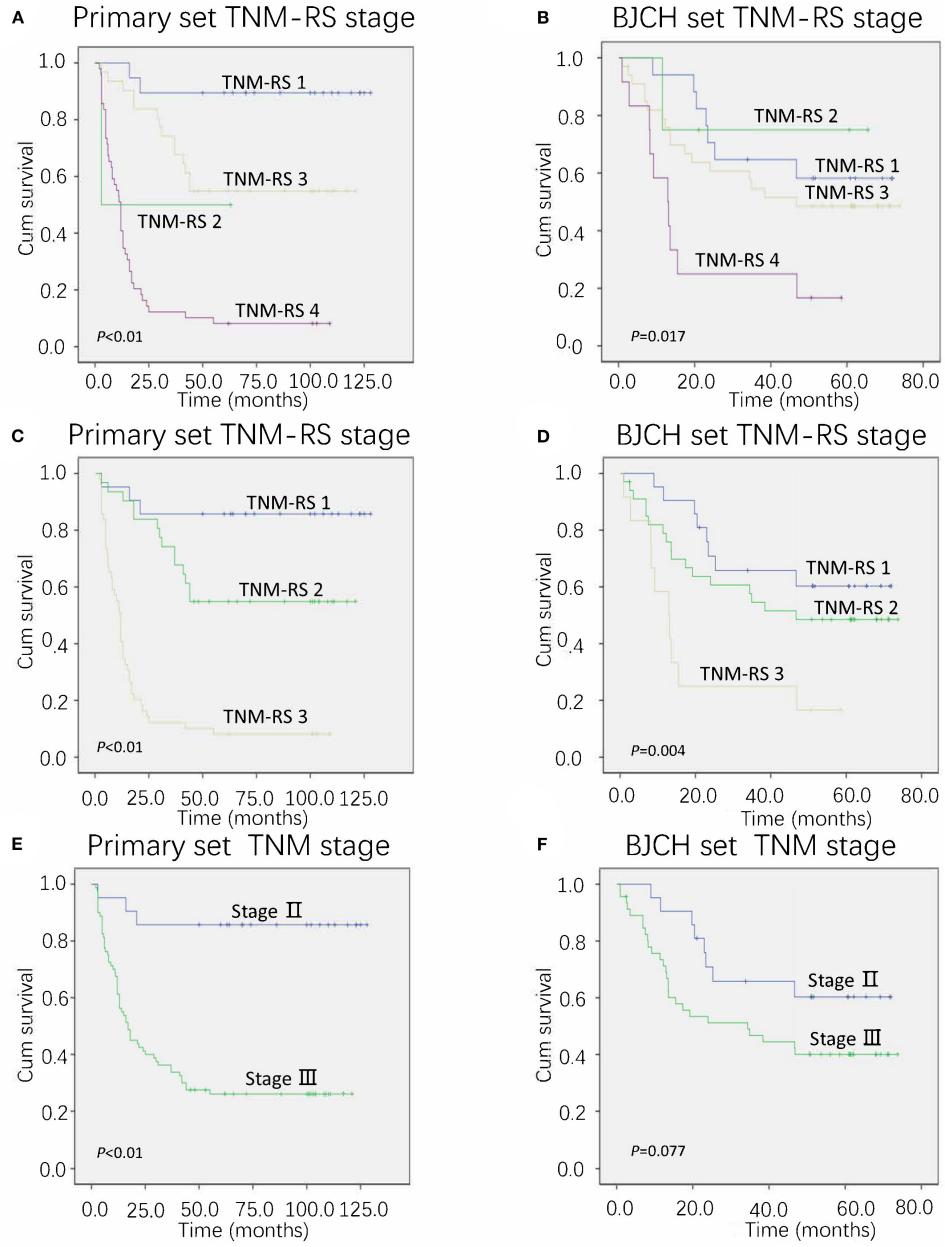
Overall survival of primary and Beijing Cancer Hospital (BJCH) sets according to different staging systems. **(A,C)** Overall survival of primary set according to tumor–node–metastasis (TNM) and risk score (TNM-RS) staging system. **(B,D)** Overall survival of BJCH set according to TNM-RS staging system. **(E)** Overall survival of BJCH set according to the 7th edition of the American Joint Committee on Cancer (AJCC) staging system. **(F)** Overall survival of BJCH set according to the 7th edition of the AJCC staging system.

Furthermore, we proposed a new stage system combining conventional TNM stage and risk score stage (TNM-RS stage system). Patients with stage II and low-risk scores were defined as TNM-RS 1, those with stage II and high-risk scores were defined as TNM-RS 2, those with stage III and low-risk scores were defined as TNM-RS 3, and those with stage III and high-risk score were defined as TNM-RS 4 (Figures 5A,B). TNM-RS 1 was no different from TNM-RS 2 in overall survival (*P* > 0.05). Thus, we categorized our patients into three groups: patients in stage II were TNM-RS 1, those in stage III and low-risk scores were defined as TNM-RS 2, and those in stage III and high-risk scores were defined as TNM-RS 3 (Figures 5C,D). TNM stage was also used to classify patients as a reference (Figures 5E,F). As shown in Figures 5C–F, the new proposed TNM-RS stage system demonstrates better discrimination compared to TNM stage alone.

**Figure 6:**
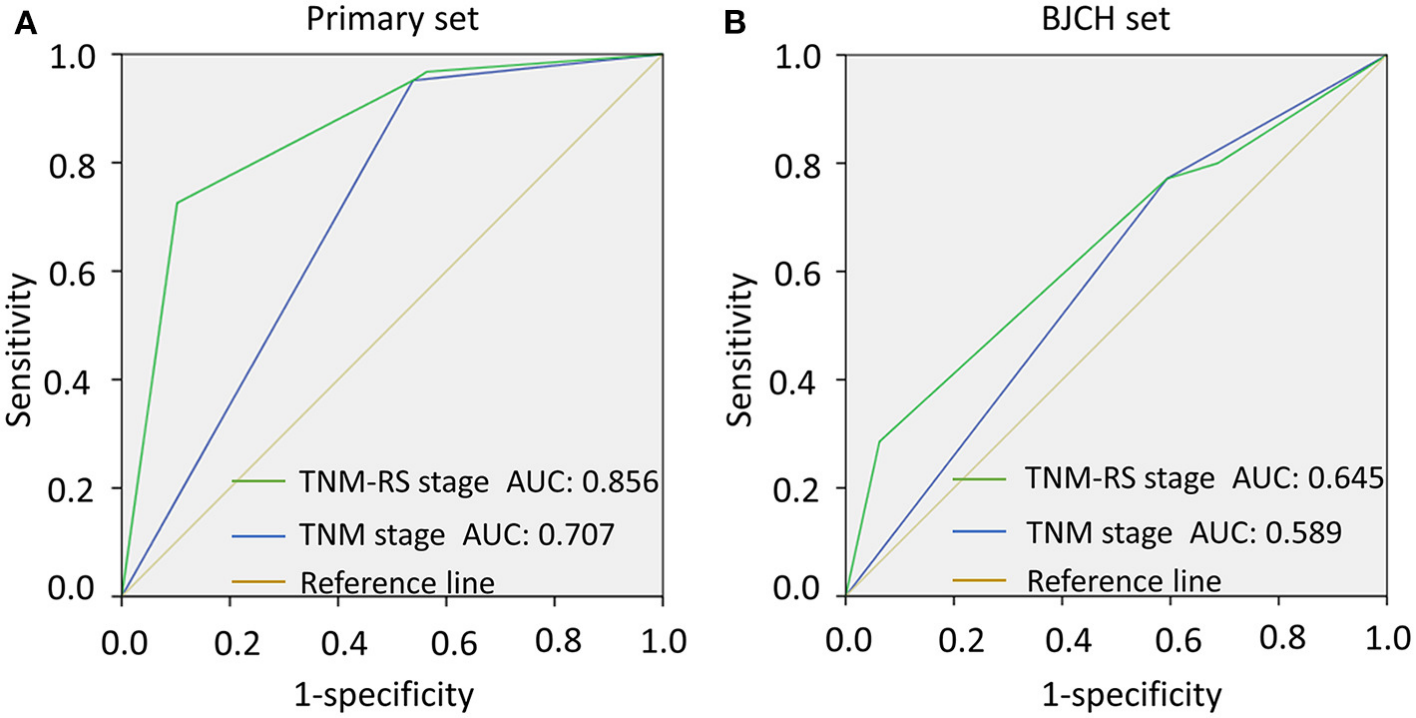
Comparison of area under curve (AUC) according to tumor–node–metastasis (TNM) stage and TNM and risk score (TNM-RS) stage. **(A)** AUCs of TNM stage and TNM-RS stage in the primary set. **(B)** AUCs of TNM stage and TNM-RS stage in the Beijing Cancer Hospital (BJCH) set.

To further verify reliability of the TNM-RS stage system, ROCs and AIC methods were performed in both primary and BJCH sets (19). TNM-RS stage system had a higher AUC (primary set, 0.856 vs. 0.707; BJCH, 0.645 vs. 0.589) and smaller AIC index (primary set, 457.2 vs. 504.4; BJCH set, 267.8 vs. 270.8) (Figures 6A,B).

### Signal Pathway Analysis of Classifier D Targeted Genes

To reveal the possible regulation mechanisms of these three miRNAs in the survival of LAGC patients, we integrated the predicted target genes of the above miRNA-related databases. An overlap of 34 (2%), 881 (9.3%), and 3 (100%) predicted genes were found for miR-132, miR-548a-3p, and miR-1826, respectively (Figure S2). Signal pathway analysis showed that target genes regulated by miR-548a-3p and miR-1826 were both involved in focal adhesion and Wnt pathways. We also found that miR-1826 regulated genes involved in phosphatidylinositol-3-kinase (PI3K)-Akt, Ras, and transforming growth factor beta (TGF-β) pathways. miR-548a-3p regulated genes involved in cell cycle, mitogen-activated protein kinase (MAPK), and Notch pathways. In addition, miR-132 was found to regulate genes involved in positive regulation of protein serine/threonine kinase activity. Gene annotation analysis results showed that genes regulated by these three miRNAs were involved in critical biological process associated with cancers (Table 6 and Figures S3, S4).

**Table 6:** Targeted genes regulated by miR-132, miR-1826, and miR-548a-3p that are involved in signal pathways.

**miRNAs**	**Pathway name**	**Symbol**			***P* value**
miR-132	Positive regulation of protein serine/threonine kinase activity	SRC	RPTOR		0.056793
miR-1826	Focal adhesion	CTNNB1	MAP2K1	VEGFC	0.016
	PI3K-Akt signaling pathway	MAP2K1	VEGFC		0.025
	Ras signaling pathway	MAP2K1	VEGFC		0.014
	TGF beta signaling pathway	MAP2K1			0.042
	WNT signaling pathway	CTNNB1			0.042
miR-548a-3p	Cell cycle	CCNH	CREBBP	GSK3B	0.016
		RB1	SKP2	SMAD4	
		STAG2	YWHAZ		
	Focal adhesion	ACTB	COL5A1	IGF1	0.007
		LAMA4	PTEN	VEGFA	
	MAPK signaling pathway	CACNA1C	MAP3K1	PRKCA	0.038
		RAC1	TGFB2		
	Notch signaling pathway	ADAM10	EP300	GATA3	0.007
		NOTCH2	PSEN1		
	Wnt signaling pathway	APC	AXIN2	CAMK2A	4.68e-4
		FZD10	PLCB1	PPP3CA	
		SMAD4			

## Discussion

Compared to a single miRNA, a cluster of biomarkers is a better prognostic tool with much higher sensitivity, specificity, and accuracy. For example, a seven-miRNA signature was identified as a specific biomarker for GC by Li et al. (20). Similarly, Zhang et al. confirmed a predictive value of hsa-miR-375 and hsa-miR-142-5p in recurrence risk (21). However, these miRNAs were from paraffin-embedded tissue blocks and fresh frozen GC tissues, respectively. It is often difficult to acquire desired miRNAs from tissues. Therefore, the nature of invasion, discomfort, and inconvenience limits its application in clinical practice.

Recently, serum miRNAs have been studied extensively compared with conventional tissue miRNAs due to the non-invasiveness of their collection, their stability, and convenience (22, 23). The roles of serum-based miRNA in cancer diagnosis have also been largely demonstrated (22, 23). However, its roles in cancer prognosis have not been adequately evaluated, especially in GC.

In this study, we first established and validated a novel serum signature based on three miRNAs (miR-132, miR-1826, and miR-548a-3p) for LAGC. This model demonstrated better discrimination in survival prediction. Moreover, we revealed that this signature is a powerful and independent prognostic classifier by both internal and external validation.

Previously, expression of miR-132 and miR-1826 in tissue was shown to play a pivotal role in inhibiting cancer progression (24, 25). Conversely, miR-548a-3p was reported to be associated with human lymphoblastoid cell line proliferation and apoptosis as an oncogene (26). These findings are consistent with the currently study. The release mechanism of these miRNAs may be related to microvesicles or exosomes generated by tumors (27, 28).

Furthermore, we established a new staging system (TNM-RS stage) by combining conventional TNM stage and risk score stage. Interestingly, we found that patients with various clinical outcomes were better stratified using these methods. The novel TNM-RS stage exhibited better predictive efficiency than did TNM stage alone. Large variations in clinical outcomes were commonly observed when categorizing patients according to the conventional staging system (29, 30), implying that conventional clinical features are inadequate for prognostic prediction. For the first time, we reveal that a serum miRNA signature has the potential to be a novel complement to the conventional TNM staging system.

Despite their critical role, only a limited number of miRNAs are known to regulate specific target genes (31). The definite gene targets and mechanisms of the classifiers to predict LAGC remain enigmatic. It is reported that downregulation of miR-132 indicates poor survival in colorectal cancer as a result of hypermethylation (32). Similarly, miR-1826 was reported to play an important role as a tumor suppressor through CNTTB1 and MEK1 in von Hippel–Lindau (VHL)-inactivated renal and bladder cancers (33, 34). miR-548a-3p was also found to be associated with protein kinase cascades, lymphocyte proliferation, and apoptosis in human lymphoblastoid cell lines (26). In the current study, we found that these miRNAs might be involved in critical cancer-associated pathways, such as the cell cycle, Wnt, focal adhesion, and Notch. These relative oncogenic mechanisms offer us a new perspective of miRNAs' role in the pathogenesis of LAGC. More integrated investigation of the definitive targets and regulatory mechanisms of miRNAs will help us understand the disease better and guide individualized treatment.

In conclusion, our findings show that a three miRNA-based serum classifier effectively predicts survival in LAGC. This easy-to-use prognostic tool successfully categorizes patients into high- and low-risk groups. Meanwhile, a combination of the miRNA signature and conventional staging system predicts patient prognosis more accurately for LAGC. Given the characteristic of non-invasiveness and easy detection, our serum miRNA signature could further be used in monitoring therapeutic response by tracking dynamic expression profiling and personalized treatment.

## Synopsis

Current staging systems based on clinicopathological factors are inadequate in classifying patients with advanced gastric cancer (AGC). In this study, a novel serum-based microRNA signature was established for the survival prediction of the patients with AGC. A combination of the microRNA signature and conventional staging system could predict patients' prognosis more accurately. The more efficient risk stratification for AGC patients underwent surgery was of great importance for more personalized adjuvant treatment subsequently.

## Data Availability Statement

The data that support the findings of this study are available on reasonable request from the corresponding authors (Aiwen Wu and Dazhi Xu). The data are not publicly available due to restrictions of informed consent.

## Ethics Statement

The studies involving human participants were reviewed and approved by Ethical committees of Sun Yat-sen University Cancer Center. The patients/participants provided their written informed consent to participate in this study.

## Author Contributions

DX and AW: substantial contributions to the conception and design. DX, QG, JZ, SC, and JL: development of methodology. SC, JL, QG, and JZ: acquisition of data. SC and JL: analysis and interpretation of data. SC and DX: draft of the article. All authors are critical revision of the article and final approval of the version.

## Conflict of Interest

The authors declare that the research was conducted in the absence of any commercial or financial relationships that could be construed as a potential conflict of interest.

## Supplementary Material

Figure S1 Kaplan-Meier curves of stage II GC patients indicate the relationship between risk score and clinical outcome. **(A)** Survival curves of low-risk scores compared with high-risk scores in the primary set. **(B)** Survival curves of low-risk scores compared with high-risk scores in the BJCH set.

Figure S2 Venn diagrams of the number of target genes regulated by miR-132, miR-1826, and miR-548a-3p in databases TargetScan, microRNAdb, miTARbase, and miRPathDB.

Figure S3 Molecular signal pathway networks consisted of target genes that are regulated by miR-132 and miR-1826.

Figure S4 Molecular signal pathway networks consisted of target genes that are regulated by miR-548a-3p.

Figure S5 The flow chart illustrating the process of this study.

Table S1 Clinicalpathological features of the 12 cases for discovery. AJCC, American Joint Committee on Cancer; TNM, Tumor-Node-Metastasis; OS, Overall survival.

Table S2 Sequence of primers.
