# Supplementary figures and images for: A 3-MicroRNA Signature Identified From Serum Predicts Clinical Outcome of the Locally Advanced Gastric Cancer
Source: Front Oncol. 2020 Jun 19;10:565. doi: 10.3389/fonc.2020.00565 (PMC7323914; doi:10.3389/fonc.2020.00565)

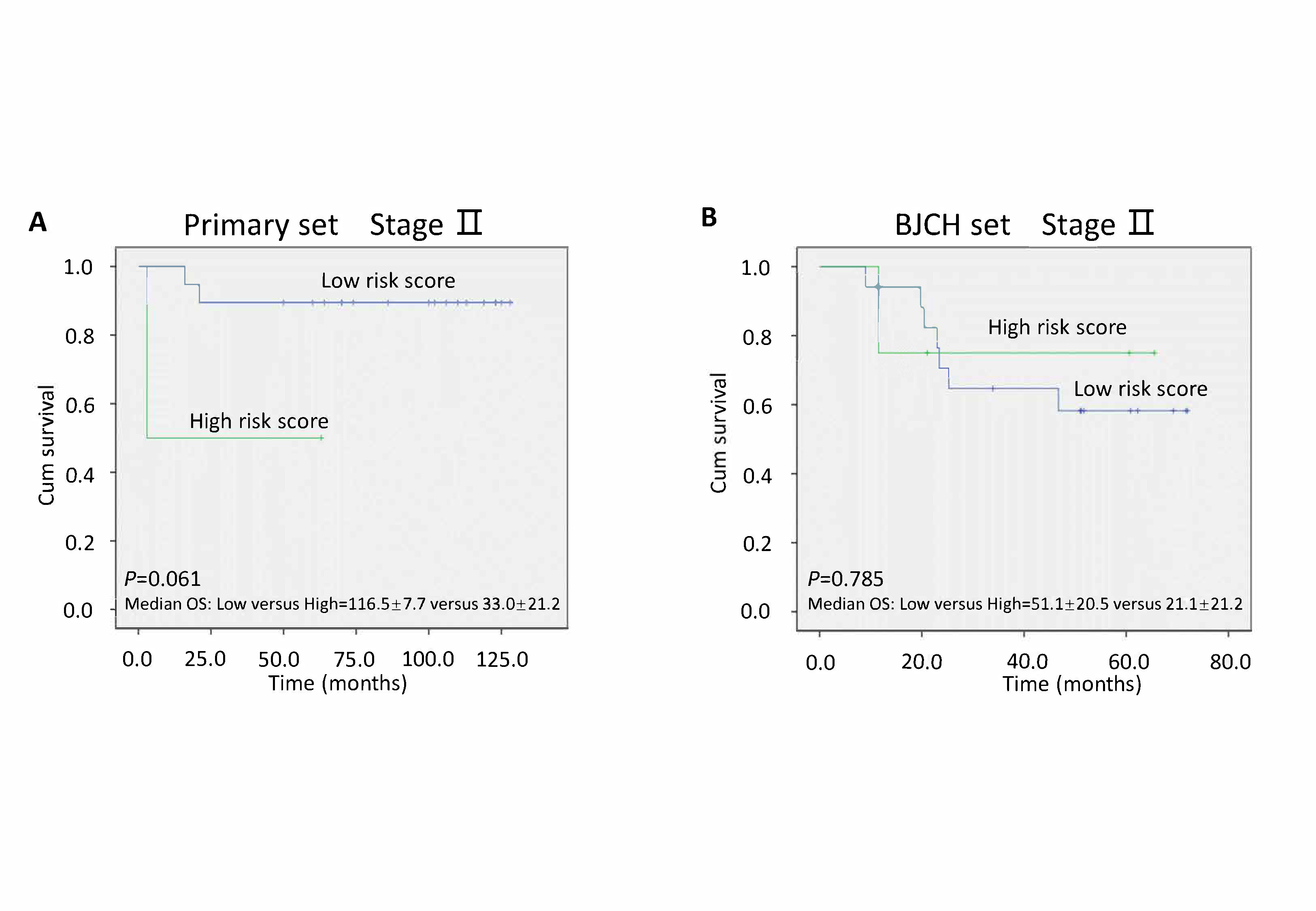

Supplement: Figure S1 — Kaplan-Meier curves of stage II GC patients indicate the relationship between risk score and clinical outcome. (A) Survival curves of low-risk scores compared with high-risk scores in the primary set. (B) Survival curves of low-risk scores compared with high-risk scores in the BJCH set. [file Image_1.tif]

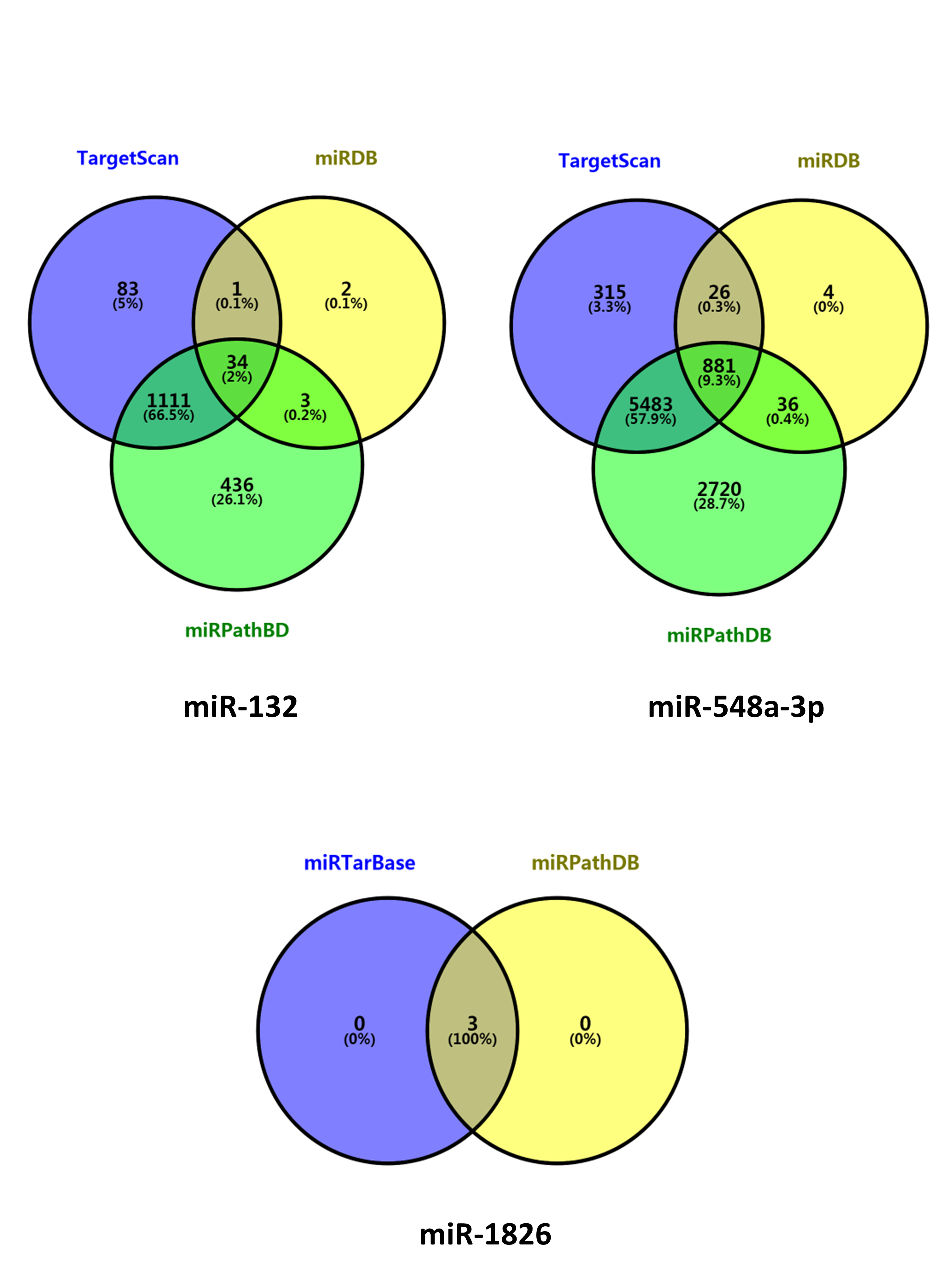

Supplement: Figure S2 — Venn diagrams of the number of target genes regulated by miR-132, miR-1826, and miR-548a-3p in databases TargetScan, microRNAdb, miTARbase, and miRPathDB. [file Image_2.tif]

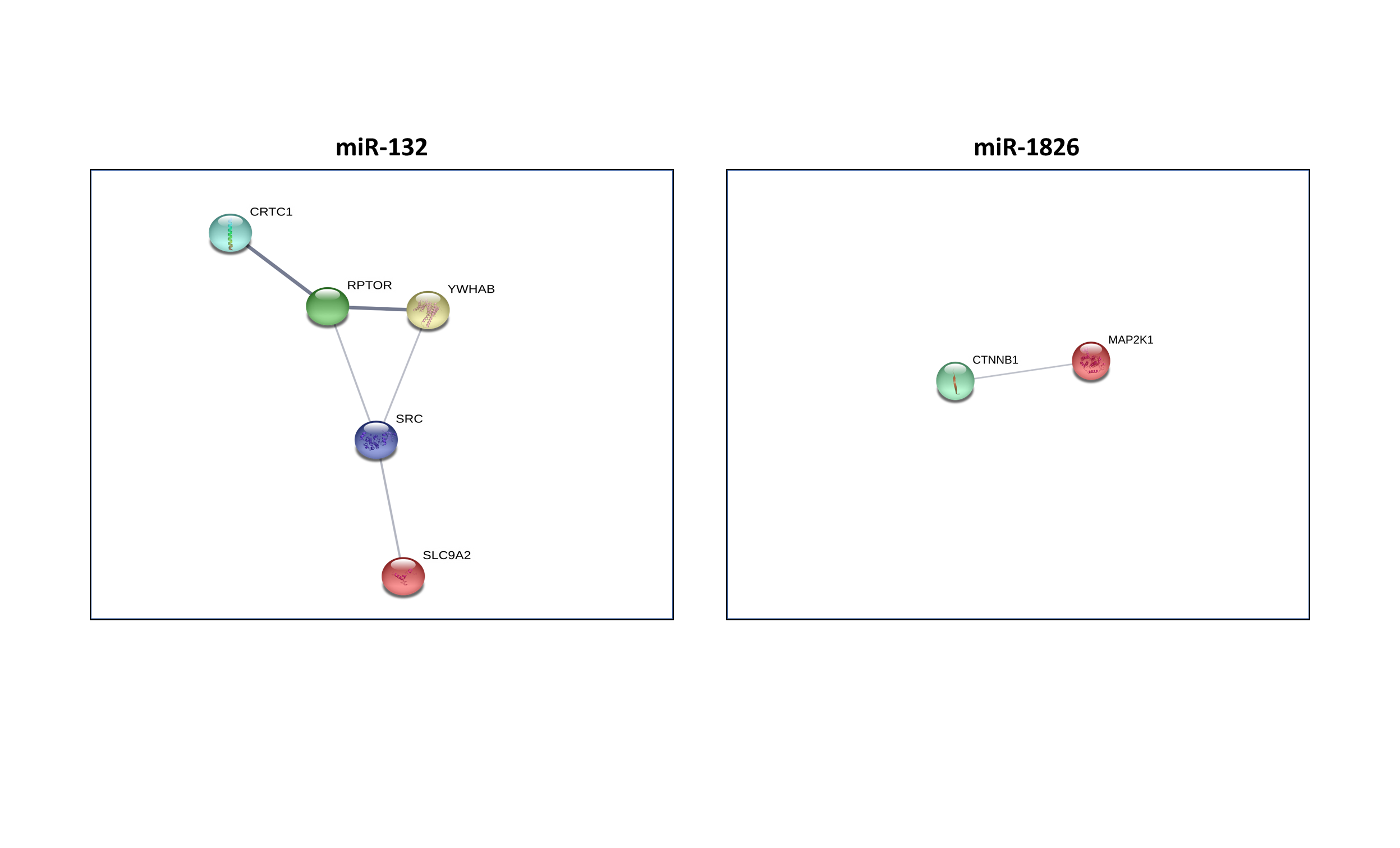

Supplement: Figure S3 — Molecular signal pathway networks consisted of target genes that are regulated by miR-132 and miR-1826. [file Image_3.tif]

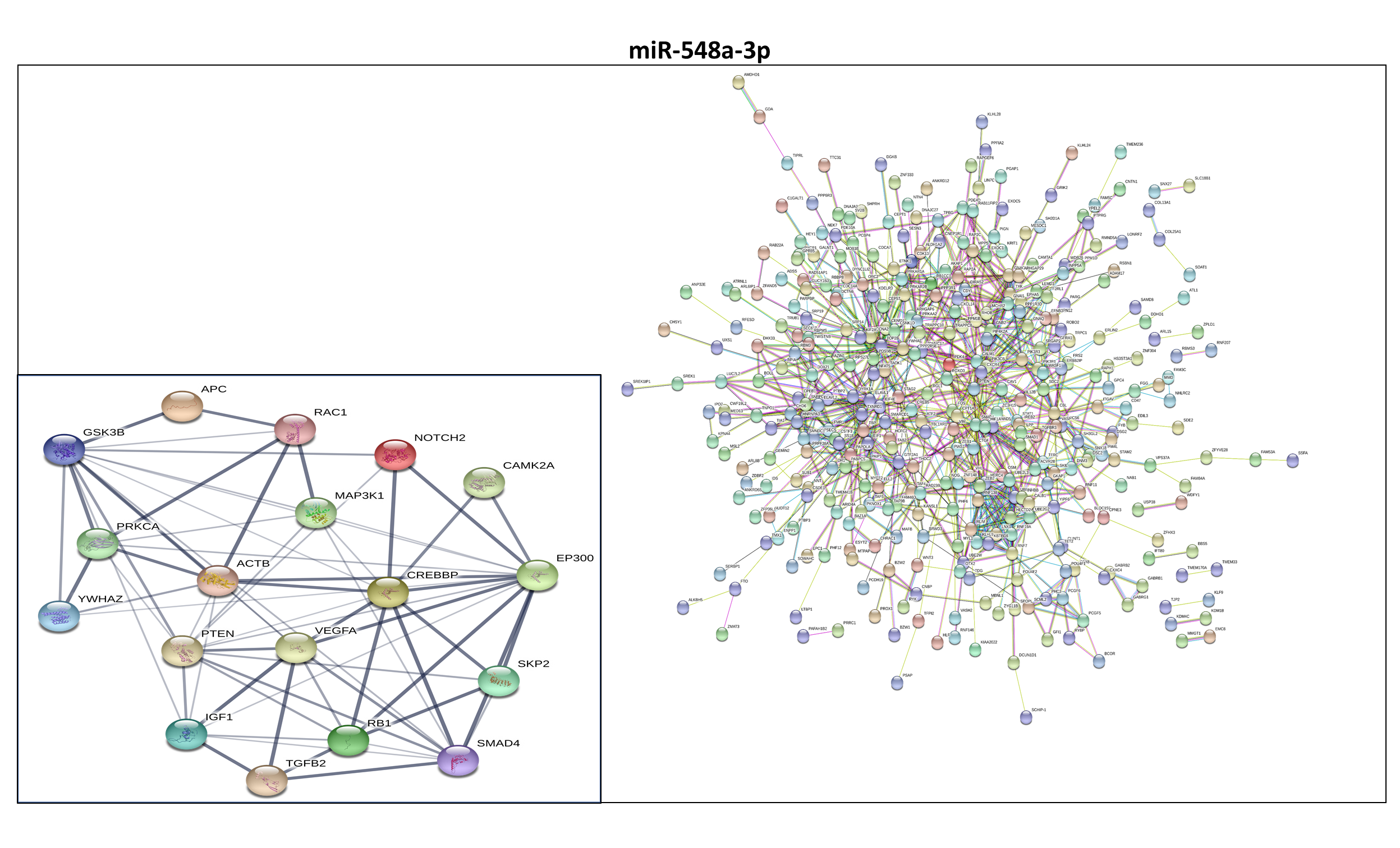

Supplement: Figure S4 — Molecular signal pathway networks consisted of target genes that are regulated by miR-548a-3p. [file Image_4.tif]

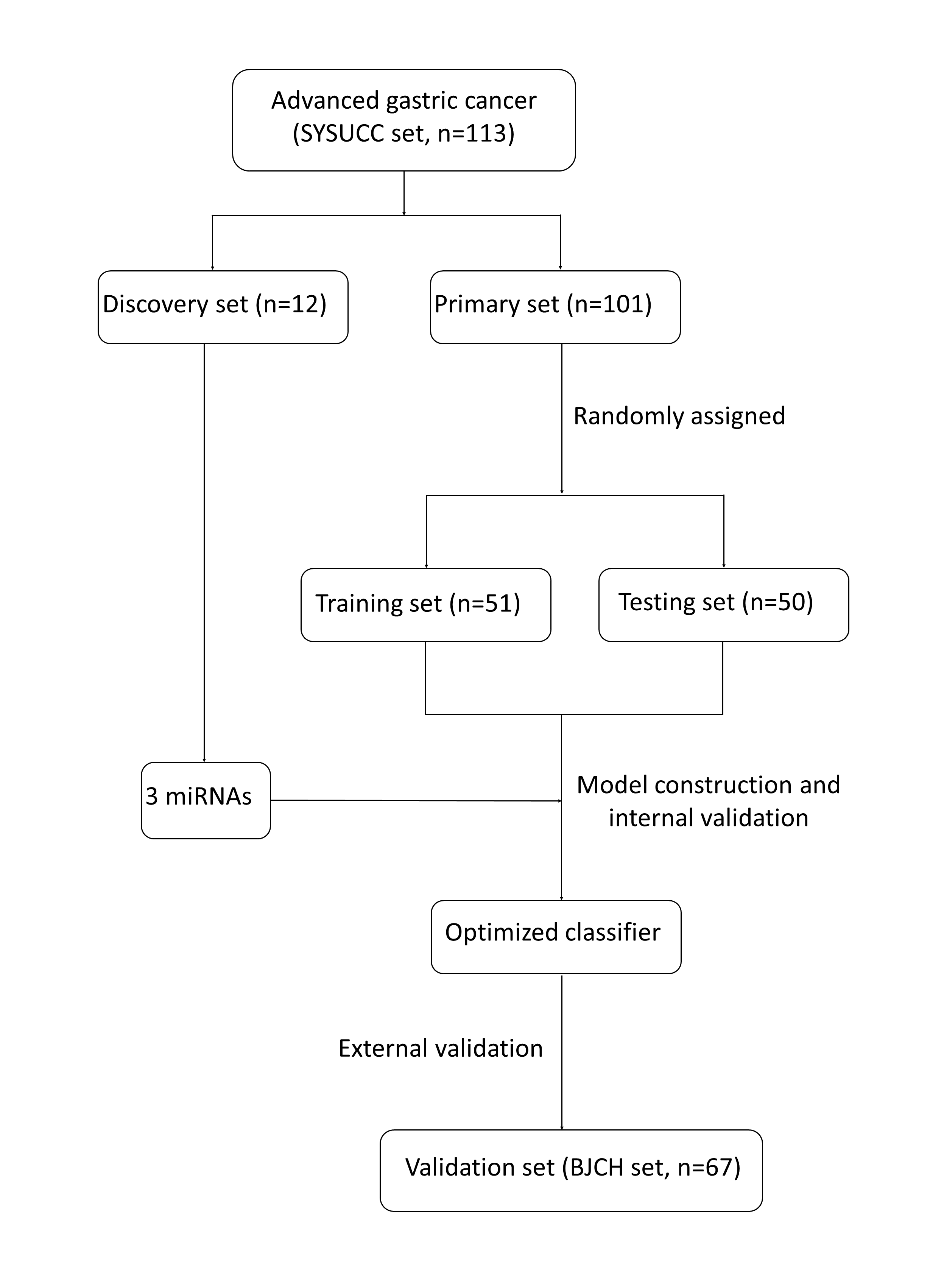

Supplement: Figure S5 — The flow chart illustrating the process of this study. [file Image_5.tif]
