# Supplementary material for: A 3-MicroRNA Signature Identified From Serum Predicts Clinical Outcome of the Locally Advanced Gastric Cancer
Source: Front Oncol. 2020 Jun 19;10:565. doi: 10.3389/fonc.2020.00565 (PMC7323914; doi:10.3389/fonc.2020.00565)
Supplement: Table S2 — Sequence of primers. [file Table_2.docx]

**Table S2. Sequence of primers**

| Name | Sequence (5’-3’) |
| --- | --- |
| microRNA-1826-Forward | GCATTGATCATCGACACTTCGA |
| microRNA-1826-Reverse | GTGCAGGGTCCGAGGT |
| microRNA-132-3p-Forward | CGGGCTCCACATCTGTTG |
| microRNA-132-3p-Reverse | CAGCCACAAAAGAGCACAAT |
| microRNA-548a-3p-Forward | CGACTCAGTCTGATGCCGTTTTTC |
| microRNA-548a-3p-Reverse | CAGCCACAAAAGAGCACAAT |
| Homo U6-Forward | CTCGCTTCGGCAGCACA |
| Homo U6-Reverse | AACGCTTCACGAATTTGCGT |
